# Transvaginal natural orifice transluminal endoscopic surgery for uterosacral ligament suspension: pilot study of 35 cases of severe pelvic organ prolapse

**DOI:** 10.1186/s12893-021-01280-6

**Published:** 2021-06-08

**Authors:** Zhiying Lu, Yisong Chen, Xiaojuan Wang, Junwei Li, Keqin Hua, Changdong Hu

**Affiliations:** Department of Gynecology, The Obstetrics and Gynecology Hospital of Fudan University, 128 Shenyang RD, Shanghai, 200090 China

**Keywords:** Transvaginal natural orifice transluminal endoscopic surgery, Pelvic organ prolapse, Uterosacral ligament suspension

## Abstract

**Background:**

To describe the short-term outcomes of transvaginal natural orifice transluminal endoscopic surgery (vNOTES) for uterosacral ligament suspension (USLS) in patients with severe prolapse.

**Methods:**

This was a retrospective study of patients with severe prolapse (≥ stage 3) who underwent vNOTES for USLS between May 2019 and July 2020. The Pelvic Organ Prolapse Quantification (POP-Q) score, Pelvic Organ Prolapse/Urinary Incontinence Sexual Questionnaire short form (PISQ-12) and Pelvic Floor Inventory-20 (PFDI-20) were used to evaluate physical prolapse and quality of life before and after vNOTES for USLS.

**Results:**

A total of 35 patients were included. The mean operative duration was 111.7 ± 39.4 min. The mean blood loss was 67.9 ± 35.8 ml. Statistically significant differences were observed between before and after vNOTES USLS in Aa (+ 0.6 ± 1.7 versus − 2.9 ± 0.2), Ba (+ 1.9 ± 2.2 versus − 2.9 ± 0.3), C (+ 1.5 ± 2.2 versus − 6.9 ± 0.9), Ap (− 1.4 ± 1.0 versus − 3.0 ± 0.1) and Bp (− 1.1 ± 1.4 versus − 2.9 ± 0.1) (P < 0.05 for all). The mean pre- and postoperative PFDI-20 score was 19.9 ± 6.7 and 3.2 ± 5.4, respectively, and the mean pre- and postoperative PISQ-12 score was 24.8 ± 2.3 and 38.3 ± 4.1, respectively (P < 0.05 for both). During 1–13 months of follow-up, there were no cases of severe complications or recurrence.

**Conclusions:**

vNOTES for USLS may be a feasible technique to manage severe prolapse, with promising short-term efficacy and safety. Larger studies with more patients and longer follow-up periods should be performed to evaluate the long-term efficacy and safety profile of vNOTES for USLS.

## Background

Pelvic organ prolapse (POP) is a common benign condition with a high risk of occurrence of 40–60% in elderly women [1] and a reported lifetime risk of surgical intervention of 10–20% [2]. The gold-standard surgical treatment for POP is resuspension of the pelvic anatomy by native tissue repair or mesh repair. Mesh repair is associated with high rates of surgical complications and postoperative adverse events [3]. On 16 April 2019, transvaginal repair products were withdrawn from the market by the FDA [4]. Native tissue repair has received increasing attention in reconstructive pelvic surgery. Uterosacral ligament suspension (USLS) is a commonly performed procedure to support the vaginal apex [5].

Many procedures, such as laparoscopic procedures via an abdominal approach and transvaginal procedures, have been described for USLS. However, transvaginal USLS carries up to an 11% risk of ureteral injury, which is attributed to poor visibility [6, 7]. Unger et al. performed the largest retrospective series examining transvaginal USLS and found a 4.5% risk of intraoperative ureteral kinking [8]. The abdominal approach via laparoscopy offers improved visibility and allows these vaginal complications to be prevented, resulting in better suspension than the vaginal approach [9, 10]. Houlihan et al. compared uterosacral vault suspension at the time of hysterectomy via a laparoscopic versus vaginal approach. The results showed less ureteral kinking (0 vs. 14%, p = 0.023), urinary retention (15% vs. 31%, p = 0.024), and symptomatic recurrence (24% vs. 41%, p = 0.046) in the laparoscopic group [11].

In recent years, some scholars have reported the advantages of transvaginal natural orifice transluminal endoscopic surgery (vNOTES) for USLS, which include no incisional pain and a better cosmetic outcome than laparoscopy via the abdominal approach, as well as direct visualization of key structures, such as the ureters and rectum, which is not available with the traditional transvaginal approach [12, 13]. At present, there have been few studies on this topic. Therefore, it is clinically important to thoroughly research the safety and efficacy of this procedure given the paucity of existing data.

In this study, we performed USLS for POP using the vNOTES approach. Our objective was to describe the short-term outcomes of vNOTES for USLS in patients with severe apical prolapse.

## Methods

### Patients

We retrospectively collected data on all cases of vNOTES for USLS at the Obstetrics and Gynecology Hospital of Fudan University between May 2019 and July 2020. The inclusion criteria were as follows: (1) age 25–79 years; (2) severe apical prolapse (≥ stage 3); (3) desire for preservation of coital function; (4) first surgical treatment for POP; and (5) refusal of mesh implantation. The exclusion criteria were as follows: (1) inability to tolerate surgery; (2) coagulation dysfunction; (3) severe vaginal ulcers; (4) history of severe adhesions, a fixed uterus or strong pelvic adhesions noted on pelvic examination; (5) inability to tolerate the Trendelenburg position; and (6) suspicion of gynaecological malignancy. This retrospective study was approved by our institutional review board before any data were collected (no. 2019–32).

We extracted the following information from medical records: patient demographics (age, body mass index, parity, and history of prior hysterectomy or prolapse repair) and perioperative outcomes. Perioperative data included the operative duration, blood loss, intraoperative complications (transfusion or injury), postoperative complications (infection, urinary retention, persistent pain, haematoma, constipation, dyspareunia, de novo stress urinary incontinence or deep vein thrombosis), length of postoperative hospital stay, and hospitalization costs. We also assessed the change in physical prolapse with Pelvic Organ Prolapse Quantification (POP-Q). Two validated questionnaires, the Pelvic Organ Prolapse/Urinary Incontinence Sexual Questionnaire short form (PISQ-12) and Pelvic Floor Inventory-20 (PFDI-20), were completed before and at least 3 months after vNOTES for USLS to assess the impact on quality of life. Postoperative follow-up visits were scheduled at 1 month, 3 months, 6 months, 1 year and 2 years after surgery. Any POP-Q score greater than or equal to − 1 cm was defined as recurrence [14].

### Surgical procedures

All participants underwent surgery with general anaesthesia and standard operative care. Patients treated with hysterectomy received cefuroxime preoperatively. A single-port device with four trocars (HTKD Medical, China) and nonabsorbable or delayed absorbable sutures (2–0, Ethicon LLC, America) were used.

### Transvaginal hysterectomy with vNOTES for USLS

**Fig. 1:**
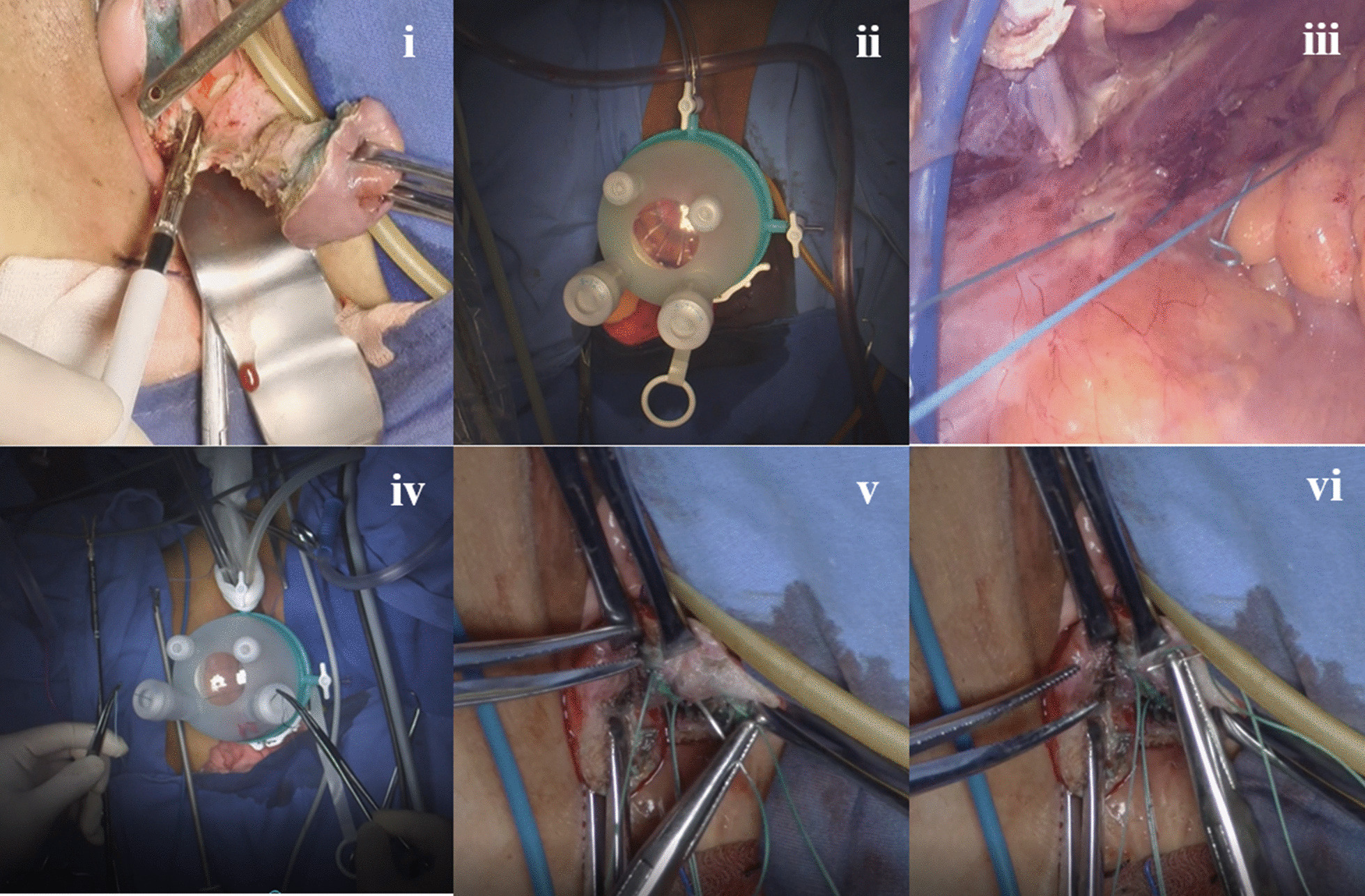
Steps of transvaginal hysterectomy with transvaginal natural orifice transluminal endoscopic surgery (vNOTES) for uterosacral ligament suspension (USLS). i: Transvaginal hysterectomy. ii: Installation of the single-port platform. iii: Placement of one nonabsorbable suture around the intermediate portion of the USL at the level of the ischial spine bilaterally, for a total of 4 stitches (right USL shown). iv: Slight tugging of the sutures to confirm correct placement. v: Attachment of the above nonabsorbable sutures to the ipsilateral cardinal ligament stump (white arrow). vi: Attachment of the above nonabsorbable sutures to the pubocervical fascia (white arrow) of the anterior vaginal wall

This surgical procedure was originally described by Lowenstein et al. in 2019 [12]. In this study, the key steps were as follows (Fig. 1): Step 1: A routine transvaginal hysterectomy was performed (Fig. 1i). Step 2: The lap protector was placed from the vaginal orifice to the pelvic cavity through the vaginal vault, and then, the single-port platform was established (Fig. 1ii). Step 3: The ureters and uterosacral ligaments (USLs) were identified under single-port laparoscopy. If the ureter was found to be very close to the ipsilateral uterosacral ligament, a peritoneal release incision was performed to avoid kinking of the ureter. Step 4: One nonabsorbable suture was placed around the intermediate portion of the USL at the level of the ischial spine bilaterally, for a total of 4 stitches (Fig. 1iii). The sutures were then tugged slightly to confirm correct placement (Fig. 1iv). Step 5: The pelvic cavity was washed with normal saline, the single-port platform was removed, and the peritoneum was closed. Step 6: The above nonabsorbable sutures were attached to the ipsilateral cardinal ligament stump (Fig. 1v) and the pubocervical fascia of the anterior vaginal wall (Fig. 1vi). Step 7: Then, the above nonabsorbable sutures were attached to the vaginal cuff and tied, suspending the vaginal cuff by the ligaments.

### Fertility preservation with vNOTES for USLS

**Fig. 2:**
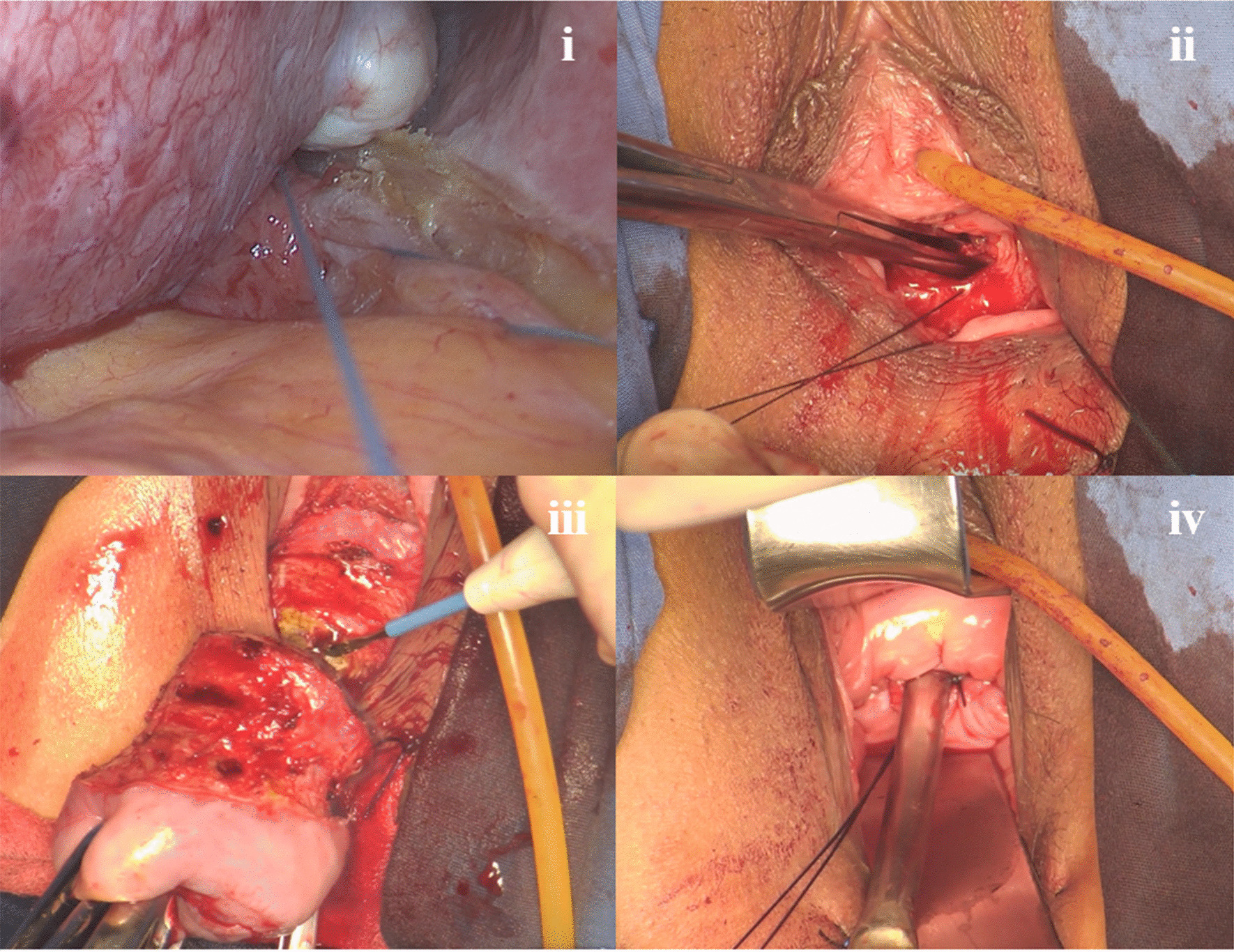
Key steps of fertility-preserving or uterine-preserving vNOTES for USLS. i: Placement of one nonabsorbable suture around the intermediate portion of the USL at the level of the ischial spine bilaterally, for a total of 4 stitches (left USL shown). ii: Attachment of the above nonabsorbable sutures to the cervix. iii: Partial excision of the cervix. iv: Reconstruction of the residual cervix

The single-port platform was placed from the vaginal orifice to the pelvic cavity through the posterior fornix. In these cases, apical support was achieved by suturing the USLs, shortening the ligaments (as mentioned above) (Fig. 2i) and reinforcing their attachment to the cervix (Fig. 2ii).

### Uterine preservation with vNOTES for USLS

In patients who desire preservation of the uterus without a requirement for fertility, part of the cervix was excised according to the degree of cervical extension (Fig. 2iii), and finally, the residual cervix was reconstructed (Fig. 2iv). The other procedures were the same as those in fertility-preserving USLS.

Anterior or posterior colporrhaphy is necessary when the anterior or posterior compartment reaches stage II prolapse. Perineal body repair was performed at the discretion of the operating surgeon such that the width of the vaginal orifice was more than that of 3 fingers at the end of the procedure. Anti-incontinence surgery was allowed according to the preoperative presence of stress urinary incontinence (SUI). The placement of a tension-free midurethral sling (MUS) was performed in patients who were willing to undergo mesh placement. If not, urethral folding was performed. Gauze was inserted into the vagina for compression.

After surgery, routine postoperative care was provided. Cefuroxime was administered once postoperatively in patients treated with hysterectomy. All patients were then followed clinically; the longest follow-up duration was 13 months.

### Statistical analysis

Data collection and statistical analyses were performed using IBM SPSS Statistics 24.0 software (IBM Corp., Armonk, New York, USA). All variables are presented as the mean and standard deviation (SD) or n and percentage (%). Continuous variables were compared by Student’s t test. A P value < 0.05 was considered statistically significant.

## Results

In total, 35 patients underwent vNOTES for USLS; in one patient, vNOTES sacrocolpopexy was planned, but the patient was converted to vNOTES for USLS because of a suspected malignant lesion on the surface of the colon. The patient characteristics are shown in Table 1. One patient had previous undergone hysterectomy for POP without any suspension. Thirty-three patients (94.3%) had stage III prolapse, and the other two patients (5.7%) had stage IV prolapse.

**Table 1:** Patient characteristics

Variable	Values
Number	35
Age (years)	53.7 ± 11.4
BMI (kg/m^2^)	24.0 ± 3.3
Parity	1.6 ± 0.8
Diabetes mellitus	1 (2.9)
Smoker	0
Prior hysterectomy	1 (2.9)
Prior prolapse repair	1 (2.9)
Prolapse stage	
Anterior	2.5 ± 0.7
Apical	2.6 ± 0.7
Posterior	1.4 ± 0.6
Baseline POP-Q stage	
III	33 (94.3)
IV	2 (5.7)

*BMI* body mass index, *POP-Q* Pelvic Organ Prolapse Quantification

Values are shown as the mean ± standard deviation or n (%)

Table 2 shows the perioperative outcomes and complications. Twenty patients (57.1%) underwent hysterectomy, 2 patients (5.7%) required fertility preservation, and 12 patients (34.3%) required uterine preservation. Three patients underwent concomitant MUS surgery, and two underwent urethral folding. Twenty-four patients underwent anterior colporrhaphy, 24 patients underwent posterior colporrhaphy, 24 patients underwent perineal body repair, 2 patients underwent ovarian cystectomy, 10 patients underwent salpingo-oophorectomy, 2 patients underwent salpingectomy, and 1 patient underwent resection of a suspected malignant lesion on the surface of the colon. The mean operative duration (calculated from anaesthesia to the end of surgery, including other concurrent surgeries) was 111.7 ± 39.4 min. The mean blood loss was 67.9 ± 35.8 ml. The mean length of postoperative hospital stay was 3.7 ± 1.1 days. The mean hospitalization cost was 3408.9 ± 592.6 dollars.

**Table 2:** Perioperative and short-term outcomes (n = 35)

Variable	Values
Intraoperative hysterectomy	20 (57.1)
Preservation of fertility	2 (5.7)
Preservation of uterus	12 (34.3)
Concurrent surgery	
Anti-incontinence surgery	5 (14.3)
MUS surgery	3 (8.6)
Urethral folding	2 (5.7)
Anterior colporrhaphy	24 (68.6)
Posterior colporrhaphy	24 (68.6)
Perineal body repair	24 (68.6)
Ovarian cystectomy	2 (5.7)
Salpingo-oophorectomy	10 (28.6)
Salpingectomy	2 (5.7)
Resection of a suspected malignant lesion on the surface of the colon	1 (2.9)
Operative duration (min)	111.7 ± 39.4
Blood loss (ml)	67.9 ± 35.8
Postoperative hospital stay (days)	3.7 ± 1.1
Hospitalization cost (USD)	3408.9 ± 592.6
Overall complications	3
Patients-related complications	3 (8.6)
Intraoperative complications	
Transfusion	0
Injury	0
Postoperative complications	
Infection	1 (2.9)
Urinary retention	1 (2.9)
Persistent pain	0
Haematoma	0
Constipation	1 (2.9)
Dyspareunia	0
De novo SUI	2 (5.7)
Deep vein thrombosis	0
Follow-up duration (months)	3.9 ± 3.8 (1–13)
Recurrence	0

Values are shown as the mean ± standard deviation (range) or n (%)

There were no cases of intraoperative blood transfusion or injury (Table 2). In total, 3 postoperative complications occurred in 3 patients (8.6%). One patient (2.9%) developed a urinary tract infection, tested positive for *Escherichia coli*, and was cured after antibacterial treatment. One patient (2.9%) developed postoperative urinary retention; this patient underwent catheterization for urine drainage and was cured after 3 days of acupuncture treatment. One patient (2.9%) suffered from constipation, which was relieved with a glycerine enema. There were no cases of de novo SUI, deep vein thrombosis, persistent pain, haematoma or dyspareunia as postoperative complications. The mean follow-up duration was 3.9 ± 3.8 (1–13) months. No patients had obvious recurrence.

Tables 3, 4 and 5 show the changes in the POP-Q, PFDI-20 and PISQ-12 scores, respectively, from preoperatively to postoperatively at the latest follow-up examination for each patient. All variables except the total vaginal length (TVL) in Table 3 showed significant improvement in physical prolapse after surgery. Seventeen patients (48.6%) were followed for more than 3 months and completed the PFDI-20 (Table 4). The Pelvic Organ Prolapse Distress Inventory 6 (POPDI-6), Colorectal-Anal Distress Inventory 8 (CRADI-8), Urinary Distress Inventory Short Form (UDI-6) and total PFDI-20 scores were significantly decreased after surgery, indicating notable alleviation of pelvic, urinary and colorectal symptoms. Nine patients (25.7%) had recovered their sexual life and completed the PISQ-12 (Table 5). The PISQ-12 score was significantly increased after surgery, indicating notable improvement in quality of sexual life.

**Table 3:** Change in POP-Q score (n = 35)

Variable	Preoperative	Postoperative	P
Aa	+ 0.6 ± 1.7	− 2.9 ± 0.2	0.000*
Ba	+ 1.9 ± 2.2	− 2.9 ± 0.3	0.000*
C	+ 1.5 ± 2.2	− 6.9 ± 0.9	0.000*
Ap	− 1.4 ± 1.0	− 3.0 ± 0.1	0.000*
Bp	− 1.1 ± 1.4	− 2.9 ± 0.1	0.000*
TVL	+ 7.4 ± 0.5	+ 7.2 ± 0.4	0.058

*POP-Q* Pelvic Organ Prolapse Quantification, *TVL* total vaginal length

Values are shown as the mean ± standard deviation. *P < 0.05 was statistically significant

**Table 4:** Change in PFDI-20 score (n = 17)

Variable	Preoperative	Postoperative	P
Quality of life			
POPDI-6	9.9 ± 3.5	0.9 ± 1.9	0.000*
CRADI-8	2.5 ± 3.0	0.7 ± 2.1	0.047*
UDI-6	7.5 ± 4.4	1.6 ± 2.8	0.000*
Total PFDI-20	19.9 ± 6.7	3.2 ± 5.4	0.000*

*POPDI-6* Pelvic Organ Prolapse Distress Inventory 6, *CRADI-8* Colorectal Anal Distress Inventory 8, *UDI-6* Urinary Distress Inventory 6, *PFDI-20* Pelvic Floor Distress Inventory 20

Values are shown as the mean ± standard deviation. *P < 0.05 was statistically significant

**Table 5:** Change in PISQ-12 score (n = 9)

Variable	Preoperative	Postoperative	P
PISQ-12	24.8 ± 2.3	38.3 ± 4.1	0.000*

*PISQ-12* Pelvic Organ Prolapse/Urinary Incontinence Sexual Questionnaire Short Form

Values are shown as the mean ± standard deviation. *P < 0.05 was statistically significant

## Discussion

In the present study, vNOTES for USLS resulted in marked improvement in both anatomical prolapse and quality of life without cases of conversion or serious peri- or postoperative complications. These results suggest that vNOTES for USLS may be a feasible technique for treating severe POP.

USLS is a classic surgical method for the treatment of middle compartment POP. vNOTES has the advantages of two traditional surgical approaches, including good exposure, no abdominal wound, and more accurate suture placement after confirmation of ischial spine localization. Studies have shown that the intermediate portion of the USL at the level of the ischial spine is the strongest. Management of the anterior vaginal wall is very important. First, anterior colporrhaphy was performed when the anterior compartment reached stage II prolapse. Lee et al. reported that concomitant anterior colporrhaphy at the time of USLS seemed to reduce the recurrence of anterior vaginal wall prolapse [15]. Second, in hysterectomy cases, the nonabsorbable sutures were attached to the ipsilateral cardinal ligament stump and the pubocervical fascia (fascia of the anterior vaginal wall); and the anterior vaginal wall was pulled up to achieve suspension of the vaginal vault and reduce recurrence. Lavelle et al. reported that USLS performed vaginally or with an abdominal approach via laparoscopy resulted in a higher prolapse recurrence rate than sacrocolpopexy for stage III prolapse [16]. vNOTES for USLS in this study presented good short-term efficacy in terms of both anatomical prolapse and quality of life, without serious complications. However, a comparative study with a larger number of patients and a longer follow-up period should be conducted.

POP is often accompanied by SUI, which is easy to identify when patients have symptoms. In this study, five patients were diagnosed with SUI by preoperative symptoms and urodynamic examination results, and all of them underwent concomitant anti-incontinence surgery. To date, there have been no cases of de novo SUI during follow-up. This is consistent with other reports in the literature; when POP is combined with SUI, prolapse repair surgery combined with anti-incontinence surgery can reduce the risk of postoperative de novo SUI [17, 18]. Occult SUI may be originally obscured by organ prolapse, and de novo SUI may appear after anatomical restoration, which is why studies with a longer follow-up period should be conducted. Postoperative urinary retention has been reported in 13–32% of patients who undergo POP repair [11, 19–21]. Houlihan et al. reported that the rate of postoperative urinary retention was 31% in vaginal USLS patients and 15% in laparoscopic USLS patients [11]. Yune et al. conducted a study to identify risk factors for postoperative urinary retention after POP repair and showed that surgical approach, age, parity, preoperative postvoid residual urine, and concomitant transvaginal anterior/posterior repair may be related factors [22]. In this study, the rate of postoperative urinary retention was 2.9%, which is inconsistent with that reported in the literature. More in-depth research is required to explore this difference.

The results of this study suggest that sexual activity was restored 3 months after surgery. However, only nine of the 17 patients recovered their sexual life. There were many factors affecting the sex lives, such as age, surgery, spouse, psychological factors, and et al. In contrast with women in Western countries, Chinese women experience a rapid decrease in sexual need with age, especially after the age of 50 [23].

At our hospital [24], vNOTES for USLS required a shorter postoperative hospital stay and lower hospitalization costs than sacrocolpopexy, which significantly reduced the national medical insurance cost of vNOTES for USLS. The procedure was less expensive because there were fewer complications and the mesh did not need to be purchased.

This study has several limitations. First, the follow-up duration was relatively short, and the sample size was limited. Another, this was an observational study with no control group and there lied in the inherent weaknesses of retrospective studies. However, to our knowledge, there are few articles to describe the surgical outcomes of vNOTES for USLS, which may provide some references.

## Conclusions

vNOTES for USLS, with promising short-term efficacy and safety, may be a feasible technique for the treatment of severe prolapse. Hence, additional studies with a larger number of patients and a longer follow-up period should be conducted.

## Data Availability

The datasets used and analyzed during the current study available from the corresponding author on reasonable request.

## Abbreviations

vNOTES: Transvaginal natural orifice transluminal endoscopic surgery
USLS: Uterosacral ligament suspension
POP-Q: Pelvic Organ Prolapse Quantification
PISQ-12: Pelvic Organ Prolapse/Urinary Incontinence Sexual Questionnaire Short Form
PFDI-20: Pelvic Floor Inventory-20
TVL: Total vaginal length
POP: Pelvic organ prolapse
SUI: Stress urinary incontinence
USL: Uterosacral ligament
